# Mineralocorticoid Deficiency as an Early Presenting Symptom of Allgrove Syndrome With Novel Mutation: A Case Report

**DOI:** 10.7759/cureus.19316

**Published:** 2021-11-06

**Authors:** Hashem A AlOmran, Fadi Busaleh, Zahra Alhashim, Manal AlHelal, Yasen Alsaleh, Aida AlJabri, Zahra A AlGhadeer, Fatimah Y AlHejji, Mousa AlMazeedi, Abdulelah M Al dandan

**Affiliations:** 1 Pediatrics, Maternity and Children Hospital, Al-Ahsa, SAU; 2 Pediatric Endocrinology, Maternity and Children Hospital, Al-Ahsa, SAU; 3 Department of Pediatrics, Ministry of the National Guard - Health Affairs, Al-Ahsa, SAU; 4 Medical Intern, King Faisal University, Al-Ahsa, SAU; 5 Medicine, King Faisal University, Al-Ahsa, SAU

**Keywords:** triple a syndrome, achalasia cardia, alacrimia, primary adrenal insufficiency, allgrove syndrome

## Abstract

Allgrove syndrome or Triple-A syndrome is a triad of achalasia, alacrimia, and adrenal insufficiency. It is a rare disease that’s only described in the literature with no known incidence rate. Atypical presentation of some cases is rarely seen, especially with monotonous symptoms. We are describing an early age of presentation with dual symptoms of Allgrove Syndrome than the triplet with novel homozygous variant at c.885G>A in the AAAS gene.

## Introduction

Allgrove syndrome (AS), also known as triple-A syndrome (achalasia, adrenal insufficiency, and alacrimia (absences of tears)), is a multi-systemic disorder that was first described in 1978 [1]. The spectrum of the disease widely varies, and it can be variably associated with autonomic nervous system dysfunction and called 4-A Syndrome [2].

AS is an autosomal recessive disorder, and has been linked with mutations in the AAAS (achalasia-addisonianism-alacrimia syndrome) gene, localized on chromosome 12q13 that encodes for the ALADIN (Alacrimia-Achalasia-Adrenal Insufficiency and Neurologic disorder) protein. The presence of this mutation confirms the diagnosis of Allgrove syndrome [3].

Allgrove syndrome is a very rare disease where the description is mostly limited to case reports in the literature and is usually reported in adults. Early diagnosis during infancy is rarely seen, and this can be due to vague confusing initial symptoms which look like other common diseases. We are reporting a phenotypically incomplete Allgrove Syndrome with early presentation in infancy [4].

## Case presentation

An 11-month-old boy presented to the pediatric emergency department (ED) at maternity and children hospital (MCH) in Al-Ahsa city in Saudi Arabia with the main complaint of poor feeding and recurrent attacks of vomiting for 3 days duration that was associated with decreased activity and lethargy. There was no history of fever, diarrhea, skin rash, or contact with the ill patient. There was a history of significant attacks with the same complaints with regular emergency department visits. 

On physical examination, his height and weight were at 25th and 10th percentiles respectively. Vital signs were as follows: temperature was 36.7°C, blood pressure was 90/56 mmHg, heart rate was 133 bpm, respiratory rate was 32 beats/m and oxygen saturation was 100% on room air. The patient was afebrile with mild tachycardia. He looked ill, lethargic, and dehydrated with sunken eyes and delayed capillary refill time. His skin was dry with scrotal and gum darkening with normal male genitalia. Systemic examination was unremarkable. 

Initial laboratory tests showed uncompensated metabolic acidosis with hyponatremic dehydration and hyperkalemia (Table 1). Due to this, the patient was resuscitated with intravenous normal saline boluses and admitted to the pediatric intensive care unit (PICU) with an initial impression of infectious gastritis to rule out sepsis. Gradual correction of hyponatremia over 48 hours and correction of hyperkalemia was carried out, but the patient continued to have fluctuating serum sodium levels after discontinuation of intravenous fluids. Moreover, based on the patient's clinical findings of skin and gum hyperpigmentation with a positive family history of cousins having adrenal insufficiency, adrenal insufficiency was suspected. Tests to determine cortisol and adrenocorticotropic hormone (ACTH) levels showed high ACTH with low cortisol levels (Table 1). Adrenal insufficiency was then confirmed through the ACTH stimulation test as there was no increment in cortisol levels (post- synacthen). As the child was also noted to have alacrimia and positive family history, Allgrove syndrome was suspected. Molecular genetic testing was confirmatory through two genes mutations which are homozygous for AAAS and heterozygous for CYP21A2. The patient was managed by intravenous hydrocortisone stress dose (50 mg/m2) and then continued on oral hydrocortisone a maintenance dose (11.1mg /body surface area ) and artificial tears. The patient improved after 3 days with no relapse of hyponatremia (Table 1) and was discharged with regular follow-up with an endocrinologist. 

**Table 1:** Laboratory Results

Complete Blood Count				
Test			Reference Range	
White blood cell count	12.8 x10^3 ^		6-17.5x10^3^mg/dL	
Hemoglobin	11.4		11.1-12.6 g/dL	
Platelets	551 x10^3^		150-350x10^3^	
Blood Chemistry Tests				
Test	Initial		Discharge	Reference Range
Serum Sodium	116		135.8	133-152 mmol/L
Serum Potassium	6		4.6	3.4-5.3 mmol/L
Serum Calcium	2.49		2.49	2.12-2.52 mmol/L
Serum Magnesium	0.95		0.83	0.74-0.99 mmol/L
Serum Chloride	78		104	98-115 mmol/L
Serum Phosphate	1.9		1.49	0.8-1.5 mmol/L
Blood glucose	70		89	74-106 mg/dL
Blood urea nitrogen	8.62		3.35	1.70-8.30 mmol/L
Creatinine	24.81		24.66	49-115 μmol/L
Venous Blood Gas				
Test	Initial		Discharge	Reference Range
PH	7.347		7.45	7.35-7.46
Partial pressure of carbon dioxide (PCO_2_)	30		37	35-45 mmHg
Bicarbonate(HCO_3_)	17.5		20	22-26 mmol/L
Hormonal Profile				
Test			Reference Range	
Adrenocorticotropic Hormone (ACTH)	373.7		5-60 pg/ml	
Cortisol level post Adrenocorticotropic Hormone (ACTH) stimulation test				
0 time	244.3	>536 nmol/L		
30 minutes	236			
1 hour	231			
Aldosterone	3.7	2-7 ng/dL		
Renin	550	4.4-46.1uIU/mL		
Thyroid-Stimulating Hormone (TSH).	7.88	0.270-4.200 uIU/ml		
Free Thyroxine (FT4)	15.51	5-15 pmol/L		
Free Triiodothyronine (FT3)	6.97	3.95-6.80 pmol/L		
Dehydroepiandrosterone sulfate (DHE-S)	0.2	3.4-124 umol/L		
Microbiology				
Test			Result	
Blood culture			Negative	
Urine culture			Negative	

Urine Electrolytes		
Electrolyte	Result	Reference range:
Urinary Sodium	92	20-60mmol/L
Urinary Potassium	7.6	12-62 mmol/L

## Discussion

Allgrove syndrome (AS) is a very rare autosomal recessive disease where its description is mostly limited to case reports in the literature. AS is characterized by a classic clinical triad of alacrimia, achalasia, and adrenal insufficiency [5]. Owing to the fact that this syndrome is underreported due to missed diagnosis, the exact incidence is still unknown [6]. Although most of the patients present during the latter half of the first decade of their life, adult-age onsets have also been reported [2,5]. Considering the widely phenotypic variability of the presentation, the initial diagnosis is often delayed. Hence, physicians should be vigilant with a high index of suspicion to bridge the significant gap between initial symptoms and the diagnosis [2,3]. As compared to others, our patient has an early age of presentation. This may be related to high penetrance, as there are preceded cases in the family.

Alacrimia is usually present in infancy and is consistently reported to be the earliest symptoms of AS with a prevalence reaching >90% of the patients affected [7]. Alacrimia itself is a rare condition and very unlikely to be an isolated finding in pediatric patients. Thus, children with alacrimia should always be thoroughly investigated for an underlying condition. This sign was not noted initially in our case as the patient was dehydrated and thought this is the reason for alacrimia [2]. Schirmer’s test aids the initial diagnosis and confirms the presence of reduced or absent tears (alacrimia). Orbital computerized tomography (CT) scans reveal the absence or atrophy of lacrimal glands. Biopsy often shows neuronal degeneration and depletion of secretory granules in the acinar cells [7,8]. 

Achalasia cardia is one of the motility disorders of the esophagus that is characterized by defective relaxation of the gastro-esophageal sphincter and absence of peristalsis within the body of the esophagus due to fibrosis and degeneration of the inhibitory myenteric plexus. Achalasia is not that common in children [2,9]. However, it occurs in up to 75% of AS cases, and patients usually present with progressive dysphagia, failure to thrive or weight loss, and repeated aspiration pneumonia [2,5]. Literature has suggested that achalasia mostly appears at adolescent or pre-adolescent age, and this is concise with our case [2]. Achalasia can remain undiagnosed for years, as its initial presentation mimicking gastro-esophageal reflux disease. Manometry is the gold standard for diagnosis. Additionally, esophagography shows narrowing in the cardio-esophageal junction and proximal dilatation, which helps in diagnosis if manometry is not available [6-7].

Pseudo-adrenal insufficiency in AS is due to adrenocorticotropic hormone (ACTH) resistance [6]. Glucocorticoid function is found to be deficient in up to 85% of the cases, whereas mineralocorticoid is affected only in 15% [2,7]. Thus, it should be kept in mind that the absence of electrolyte abnormality, mainly hyperkalemia, does not necessarily exclude the diagnosis of adrenal insufficiency. In AS, adrenal insufficiency generally presents at a pre-pubertal age. However, it often varies [5,6]. Patients can present with life-threatening events called “adrenal crisis” in the form of hypoglycemic symptoms (shivering, sweating, and fatigue), vomiting, abdominal pain, tachycardia, and refractory hypotension, which can be misdiagnosed with other shock disorders [2]. On the other hand, other patients come to the attention of clinicians due to hyperpigmentation of the skin and oral mucosa, failure to thrive, abdominal pain, and recurrent vomiting. This presentation brought concern and worries to our patient, especially with the presence of family history [5]. The diagnosis should be confirmed by measuring cortisol, ACTH levels, and cortisol increment after an ACTH stimulation test. Under normal circumstances, there should be an increment in cortisol levels after an ACTH stimulation test. This did not happen in the case of our patient which confirms the diagnosis of adrenal insufficiency (pseudo-adrenal insufficiency). On some occasions, there will be some response with the ACTH challenge test in some patients. Thus, the diagnosis of AS should not be ruled out completely in the absence of adrenal insufficiency. On the other hand, mineralocorticoid function is affected in only a minority of patients, and this was observed here as mineralocorticoid crisis (hyponatremic dehydration with hyperkalemia) as there were low levels of aldosterone and sky-high renin levels [7].

AS should be one of the differentials in case of early neurological dysfunction and developmental delay [7]. Progressive neurological impairment constitutes another important component of this syndrome with a prevalence of 60% of affected patients [9]. This usually manifests with polyneuropathy with distal distribution, autonomic dysregulation, amyotrophy, parkinsonism, ataxia, and dementia [5,9]. In comparison to other AS features, neurological involvement is considered a late phenomenon. The pathophysiology of neuropathy in AS is not clearly understood. However, it has been found that ACTH has some neuropathic effect, and its receptor gene could provide some clue and clarify the association between the three pathognomonic features of this syndrome. There is no specific parenchymal finding in brain magnetic resonance imaging (MRI) mentioned in the literature, but it has revealed focal demyelination at the medulla and inferior cerebellar peduncle [3,5].

Diagnosis of Allgrove can be clinically suspected but confirmation must be done through presence of genetic mutation on chromosome 12q13, AAAS (achalasia-addisonianism-alacrima syndrome) gene that encodes for ALADIN (Alacrimia-Achlasia-Adrenal Insufficiency and Neurologic disorder) protein [3]. Not all patients with the AAAS gene mutation present with the characteristic triad of AS, as the triad may be incomplete. However, at least 2 features are necessary to make the clinical diagnosis of so-called Double-A syndrome [2]. There is marked phenotypic variability in genetically confirmed patients. According to the previous literature, the characteristic triad of AS is found in almost two-thirds of patients, double features in one-third, and single feature alone in less than 10% [6,10]. 

There is no definitive treatment for this challenging condition. The management mainly focuses on the individual presenting signs and symptoms. Artificial tears and lubricants are used to relieve the dryness of the eyes and prevent complications such as keratopathy and corneal ulceration. Achalasia can be managed with calcium channel blockers, botulinum toxin injection, Heller’s cardiomyotomy as well as pneumatic esophageal dilatation. There is no consensus regarding the first line of treatment. However, a combined approach is usually required. Missed adrenal crisis is the leading cause of mortality among AS patients. Short-acting glucocorticoid hydrocortisone is the treatment of choice of adrenal insufficiency. However, fludrocortisone may also be required in some cases with persistent electrolyte disturbance, but the high doses of hydrocortisone provide a mineralocorticoid activity that restores electrolytes balance [2,7,11].

Our patient was found to have a novel mutation for Allgrove syndrome. Sequence analysis identified the homozygous variant c.885G>A in the AAAS gene which adds a premature stop codon, and subsequent mRNA degradation. To the best of our knowledge, the variant has not described in the literature (HGMD human gene mutation database 2020.1). Furthermore, our patient was found to have a heterozygous mutation for CYP21A2. This gene is responsible for the congenital adrenal hyperplasia secondary to 21 hydroxylase deficiency that presented in both mineralocorticoid and glucocorticoid deficiency properties [12]. The gene is found here in heterozygous pattern (only one copy). We do not know whether its co-existence may be the contributory factor that led to mineralocorticoid crisis as early presenting symptom in our patient, which is not the case in his cousin who was found to have only glucocorticoid deficiency. This might be by chance as it is common in our area. Subclinical hypothyroidism (SCH ) was also found in our patient, which is interestingly reported by Dr. Modebe in another Saudi boy with Allgrove and this finding was never reported before in another ethnicity to our best knowledge [13]. In addition to a paper published by Al-Jurrayan in 2015, it was found that about less than 2% of Allgrove syndrome patients in Saudi Arabia have adrenal insufficiency [14]. 

## Conclusions

Real numbers of Allgrove syndrome are still unrecognized due to the similarities of presenting symptoms with common pediatric problems and the variable phenotypic expression of this disease. Being attentive to minute details in history and examination, especially alacrimia and skin hyperpigmentation can sometimes be the clue for diagnosis and the way of preventing acute life-threatening adrenal crisis. 
